# In silico search, characterization and validation of new EST-SSR markers in the genus *Prunus*

**DOI:** 10.1186/s13104-016-2143-y

**Published:** 2016-07-07

**Authors:** Karim Sorkheh, Angela S. Prudencio, Azim Ghebinejad, Mehrana Kohei Dehkordi, Deniz Erogul, Manuel Rubio, Pedro Martínez-Gómez

**Affiliations:** Department of Agronomy and Plant Breeding, Faculty of Agriculture, Shahid Chamran University of Ahvaz, P.O. Box. 61355/144, Ahvaz, Iran; Department of Plant Breeding, Centro de Edafología y Biología Aplicada del Segura (CEBAS-CSIC), PO Box 164, E-30100 Espinardo-Murcia, Spain; Department of Agronomy, Faculty of Agriculture, Payame Noor University, P.O. Box. 19395-3697, Tehran, Iran; Department of Horticulture, Faculty of Agriculture, University of Ege, Bornova, 35100 Izmir, Turkey

**Keywords:** EST-SSR, SSR mining, Functional domain marker, *Prunus*, In silico analysis, Breeding

## Abstract

**Background:**

Simple sequence repeats (SSRs) are defined as sequence repeat units between 1 and 6 bp that occur in both coding and non-coding regions abundant in eukaryotic genomes, which may affect the expression of genes. In this study, expressed sequence tags (ESTs) of eight *Prunus* species were analyzed for in silico mining of EST-SSRs, protein annotation, and open reading frames (ORFs), and the identification of codon repetitions.

**Results:**

A total of 316 SSRs were identified using MISA software. Dinucleotide SSR motifs (26.31 %) were found to be the most abundant type of repeats, followed by tri- (14.58 %), tetra- (0.53 %), and penta- (0.27 %) nucleotide motifs. An attempt was made to design primer pairs for 316 identified SSRs but these were successful for only 175 SSR sequences. The positions of SSRs with respect to ORFs were detected, and annotation of sequences containing SSRs was performed to assign function to each sequence. SSRs were also characterized (in terms of position in the reference genome and associated gene) using the two available *Prunus* reference genomes (mei and peach). Finally, 38 SSR markers were validated across peach, almond, plum, and apricot genotypes. This validation showed a higher transferability level of EST-SSR developed in *P. mume* (mei) in comparison with the rest of species analyzed.

**Conclusions:**

Findings will aid analysis of functionally important molecular markers and facilitate the analysis of genetic diversity.

**Electronic supplementary material:**

The online version of this article (doi:10.1186/s13104-016-2143-y) contains supplementary material, which is available to authorized users.

## Background

The *Prunus* genus inside the family *Rosaceae* and order *Rosales* comprises more than 230 species. Recent molecular phylogenetic studies have concluded that this genus is divided into three important subgenera (*Amygdalus*, *Cerasus* and *Prunus*) including species with high economic value which produce edible drupes or seeds. Another fourth subgenus with less interest is the *Eplectocladus* including dessert almond species [1]. The annual worldwide production of main cultivated *Prunus* species exceeded 43 million metric tons in 2013, including 21.63 million tons of peach and nectarine fruits [*P. persica* (L.) Batsch] (2*n* = 2*x* = 16) and 2.91 million tons of almond kernels [*P. amygdalus* (Batsch) syn. *P. dulcis* (Miller) Webb] (2*n* = 2*x* = 16) in the subgenus *Amygdalus*; 49 million tons of sweet (*P. avium* L.) (2*n* = 2*x* = 16), sour (*P. cerasus* L.) (2*n* = 4*x* = 32) and ground (*P. fruticosa* Pall.) (2*n* = 4*x* = 32) cherry fruits in the subgenus *Cerasus*; 11.52 million tons of prune (*P. domestica* L.) (2*n* = 6*x* = 48), plum (*P. salicina* Lindl) (2*n* = 2*x* = 16), sloe (*P. spinosa* L.) (2*n* = 4*x* = 32), and cherry plum (myrobalan) (*P. cerasifera* Ehrh.) (2*n* = 2*x* = 16) fruits in the subgenus *Cerasus* section Prunus; and 4.11 million tons of apricot (*P. armeniaca* L.) (2*n* = 2*x* = 16) and mei (or Japanese apricot) (*P. mume* von Siebold and Zuccarini) (2*n* = 2*x* = 16) fruits in the subgenus *Cerasus* section Armeniaca (http://faostat.fao.org).

Simple sequence repeats (SSRs), also known as microsatellites, are short repeat motifs present in both protein coding and non-coding regions of DNA sequences. SSRs show a high level of length polymorphism due to mutations of one or more repeats. The use of SSRs as molecular markers is favorable due to their multi-allelic nature, reproducibility, high abundance, and extensive genome coverage [2]. On the other hand, Expressed sequence tags (ESTs) are single-pass sequences of cDNA classes that provide direct information of gene expression and also serve as sources of microsatellites [3]. The traditional methods of developing SSR markers from ESTs are usually time consuming and labor-intensive. Generally, processes involve genomic library construction, hybridization with the repeated units of nucleotides, and sequencing of the clones. These traditional methods have been applied in *Prunus* species in the development of SSR-ESTs in peach [4, 5], apricot [6, 7], almond [8, 9] and mei [10, 11]. The computational approach for developing SSR markers from ESTs provides a better platform than the conventional approach. EST databases store expressed sequences that are redundant, so they contain repetitive units [12]. Such computational approaches have been recently applied in *Prunus* species, albeit only in the reference peach genome [13, 14].

Expressed sequence tags sequences can be obtained from databases and assembled to develop potential SSR markers in different species even without the availability of a fully sequenced genome. Numerous tools (both standalone and web-based) are available for the mining of EST data to design EST-SSR markers on a large scale [15]. Free software and the large availability of EST data on the web allow researchers to easily perform rapid and low-cost data mining from their local systems. Tools such as crossmatch and trimmest provide non-redundant high-quality EST sequences that do not contain vector contamination or poly-A and -T tails. CAP3 can be used to assemble EST sequences with overlapping regions and produce contigs by joining sequences [16].

Expressed sequence tag databases have become particularly attractive resources for such in silico mining. EST–SSRs or genic SSRs as molecular markers can be obtained by database searches and other in silico approaches, and can be used in transferability studies as they contain conserved genic regions [17, 18]. Different assays have been performed in citrus [12, 19], coffee [20, 21], sugarcane [22], sunflower [23, 24], cereals [2, 17, 25, 26], eucalyptus [27], loblolly pine and spruce [28], *Ocimum basilicum* [18], *Quercus robur* [29], *Ricinus communis* [30], *Solanacea* [31], and *Brassica* species [32]. However, to the best of the authors’ knowledge, no assays have been performed in *Prunus* species.

Several reasons account for the high popularity of EST derived microsatellite markers (EST-SSRs). First, marker development from existing sequence data is fast, easy and economical. An appropriate search program can detect any type of SSR, whereas enrichment cloning captures only SSRs with predefined motifs. Second, given the preferential association of SSRs with the non-repetitive portion of plant genomes, they are a common component of ESTs [33]. Third, EST-SSRs are physically linked to expressed genes and therefore represent so-called “functional markers” that are of particular interest for marker-assisted selection [34]. Finally, primer target sequences residing in expressed DNA regions are expected to be relatively well conserved, thus enhancing the chance of marker transferability across taxonomic boundaries [35].

The objectives of this work included the in silico identification of EST-SSR markers, the functional domain marker analysis, the characterization using reference mei and peach genomes, and the validation across different *Prunus* species also analyzing the level of synteny among them.

## Results

### Assembly of EST sequences and frequency and distribution of EST-SSR motifs

A total of 111,788 ESTs were detected in different tissues (leaf, stem, root, etc.) of *Prunus* species. ESTs retrieved from NCBI (http://www.ncbi.nlm.nih.gov/) were mined for simple sequence repeats (SSRs), which were characterized and a subset for marker design. In addition, all SSR-containing sequences were annotated as far as possible.

The percentage of ESTs forming contigs was 98.8 %, indicating that the majority of ESTs had overlapping sequences with other ESTs, whereas only 1.2 % of sequences were unique and had no corresponding overlapping sequence. Following assembly, a non-redundant group of ESTs was assembled consisting of contigs and singletons, hereafter referred to as “assembled EST sequences.” A 68.75 % reduction in redundancy was observed, i.e., the number of ESTs was reduced by this proportion prior to SSR analysis. These data demonstrate the excessive overlapping that exists in EST sequences belonging to the same genome.

Analysis of EST-SSRs revealed dinucleotide SSRs to be the most common, at 26.31 %, with trinucleotide SSRs accounting for 14.58 % of all data. A large difference was apparent between the number of tri- and tetranucleotide SSRs. Nona- and decanucleotide SSRs made up less than 1 % of all data (Table 1). The frequency of occurrence of SSRs varied with the number of repeats for each type of SSR from di- to decanucleotides. In this analysis, repeat numbers from 5-mer to 10-mer and a separate class of >10-mer were assessed. For trinucleotide SSRs, 5-mer was the highest repeat number apparent. For repeat sizes of 6-mer to >10-mer, the frequency of dinucleotides was the highest (Table 1).

**Table 1:** Summary of SSR mining and frequency of different repeat types identified in the examined ESTs from the *Prunus* species assayed

Parameters	Values
*SSR mining*	
Total number of EST sequences examined	111,788
Total number of SSRs identified including poly-A and poly-T	55,884
Total number of SSRs after removing poly-A and poly-T	45,764
Number of sequences containing more than 1 SSR	316
*Repeat type* ^*a*^	
Dinucleotide	12,043 (26.31 %)
Trinucleotide	6675 (14.58 %)
Tetranucleotide	244 (0.53 %)
Pentanucleotide	125 (0.27 %)
Hexanucleotide	68 (0.14 %)
Heptanucleotide	15 (0.03 %)
Octanucleotide	9 (0.01 %)
Nonanucleotide	0 (0)
Decanucleotide	1 (0.002 %)

^a^ Data in parentheses is the percentage value of the repeat type

A total of 45,764 SSRs were identified from the 111,788 sequences screened. The most frequent repeats found within the UniGene sequences of *Prunus* species were dinucleotide repeats (26.31 %), followed by trinucleotide (14.58 %), tetranucleotide (0.53 %), pentanucleotide (0.27 %), hexanucleotide (0.14 %), heptanucleotide (0.03 %), octanucleotide (0.019 %), and decanucleotide (0.002 %) repeats, respectively. No nonanucleotide repeat was detected during the present study. Observed frequencies of different repeat types comprising the SSRs are summarized in Table 1.

Simple sequence repeats comprised 12 different dinucleotide repeats: (AG)n, (CT)n, (GT)n, (AT)n, (CA)n, (TA)n, (GC)n, (TC)n, (GA)n, (TG)n, (AT)n, (CG)n; 35 different trinucleotide repeats: (AAT)n, (AGA)n, (TGG)n, (GTG)n, (TCT)n, (AGG)n, (GAA)n, (TTG)n, (ATC)n, (GAA)n, (AAG)n, (TCC)n, (CGC)n, (GTC)n, (CAC)n, (GAT)n, (CAC)n, (GCC)n, (GCT)n, (GAT)n, (CAA)n, (GAT)n, (CAC)n, (TCT)n, (GAT)n, (TAG)n, (TTC)n, (ATT)n, (CTT)n, (AAT)n, (CTT)n, (AGA)n, (AGC)n, (TGT)n, (TGC)n; 2 different tetranucleotide repeats: (CACA)n and (GAAA)n; three different pentanucleotide repeats: (TGTAT)n, (TCAAA)n, and (GATGA)n; and 1 hexanucleotide (CACCAG)n and heptanucleotide (AAAAAAT)n repeat.

The most abundant repeats were (AT)n followed by (TC)n, (TA)n, (CT)n, (GA)n in dinucleotide, and (CTT)n followed by (AGC)n, (AAT)n, (TAT)n, (GAA)n, (TCT)n repeats in trinucleotide repeats. Among the trinucleotide repeats, (AAG)n, (CAG)n, (TGA)n, and (TGC)n also showed relatively higher frequencies, whereas other nucleotide repeats had almost equal frequencies.

### Distribution of SSRs in putative coding regions and UTRs

Analysis revealed a strong bias in the distribution of SSRs between coding regions and UTRs, with the increased frequency of SSRs in UTRs reflecting their roles as binding sites for proteins and regulatory elements. Further, the relative distribution of SSRs in coding regions revealed that trinucleotide SSRs were the most frequent (26.31 %), whereas octanucleotide, nanonucleotide, and decanucleotide SSRs were the least frequent. Tetra-, penta-, hexa- and heptanucleotide SSRs demonstrated intermediate frequencies of 0.53, 0.27, 0.14, and 0.03 %, respectively. In contrast, dinucleotide SSRs were the most frequent in UTRs (86.5 %). Penta- and hexanucleotide SSRs were not present in UTRs.

**Fig. 1:**
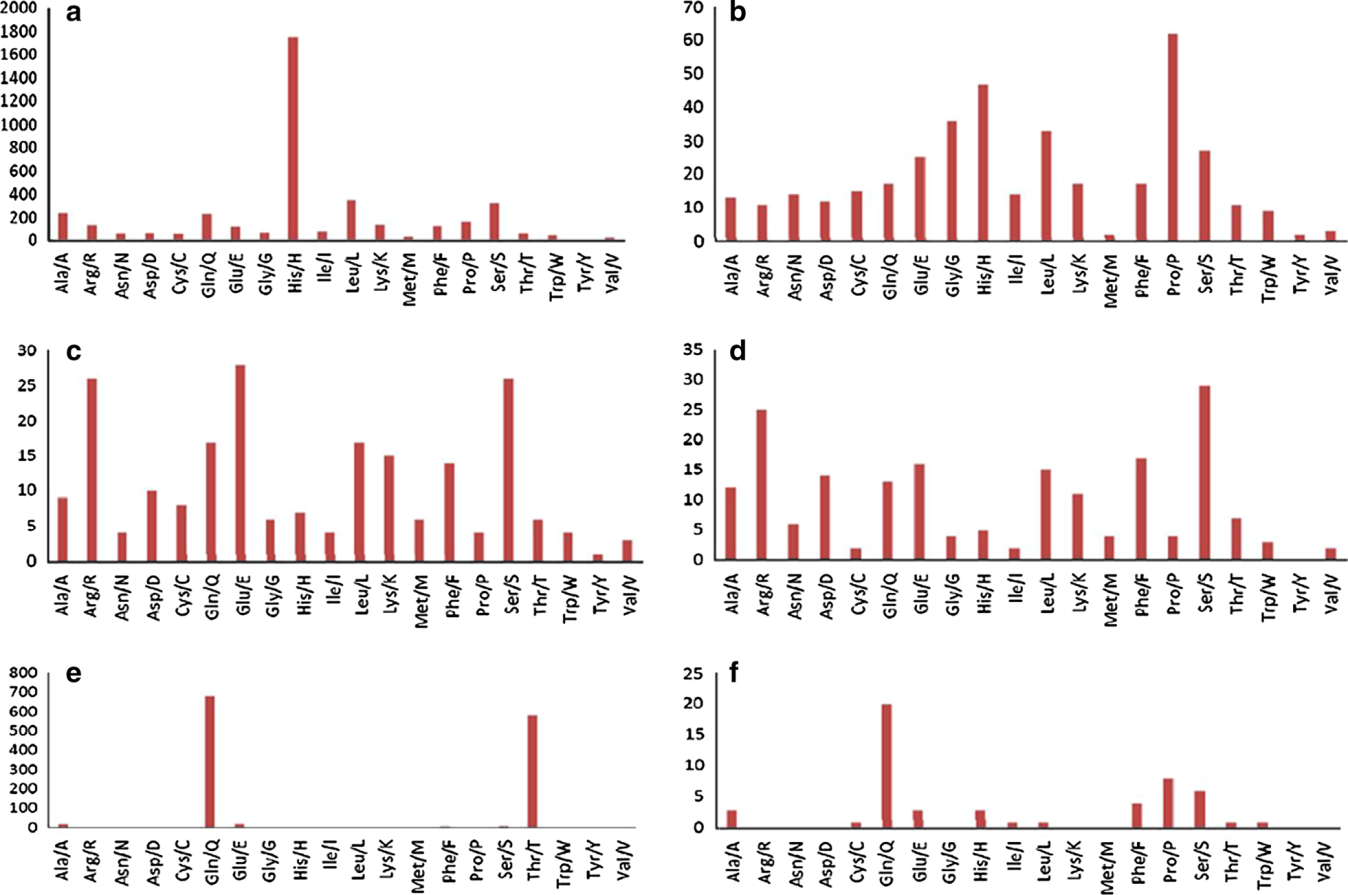
Distribution of putative encoded amino acids in *Prunus* species: *Prunus persica* (**a**), *Prunus armeniaca* (**b**), *Prunus avium* (**c**), *Prunus mume* (**d**), *Prunus dulcis* (**e**), and *Prunus cerasus* (**f**)

Each trinucleotide motif codes an amino acid that has putative roles in the biological activity of protein molecules. Of the 6270 trinucleotides identified during the present study, 27.30 % trinucleotide SSRs encoded Histidine, 14.69 % encoded Glutamine, 10.05 % Threonine, and 6.40 % Serine. However, the distribution of putative encoded amino acids differed according to the *Prunus* species assayed (Fig. 1).

Grouping of putative encoded amino acids based on their polar and non-polar nature revealed 80.66 % of amino acids to be in polar nature, and 19.33 % non-polar. This trend was consistent across all *Prunus* species assayed (Additional file 1: Fig. S1).

### Functional domain marker analysis of SSR-ESTs

**Fig. 2:**
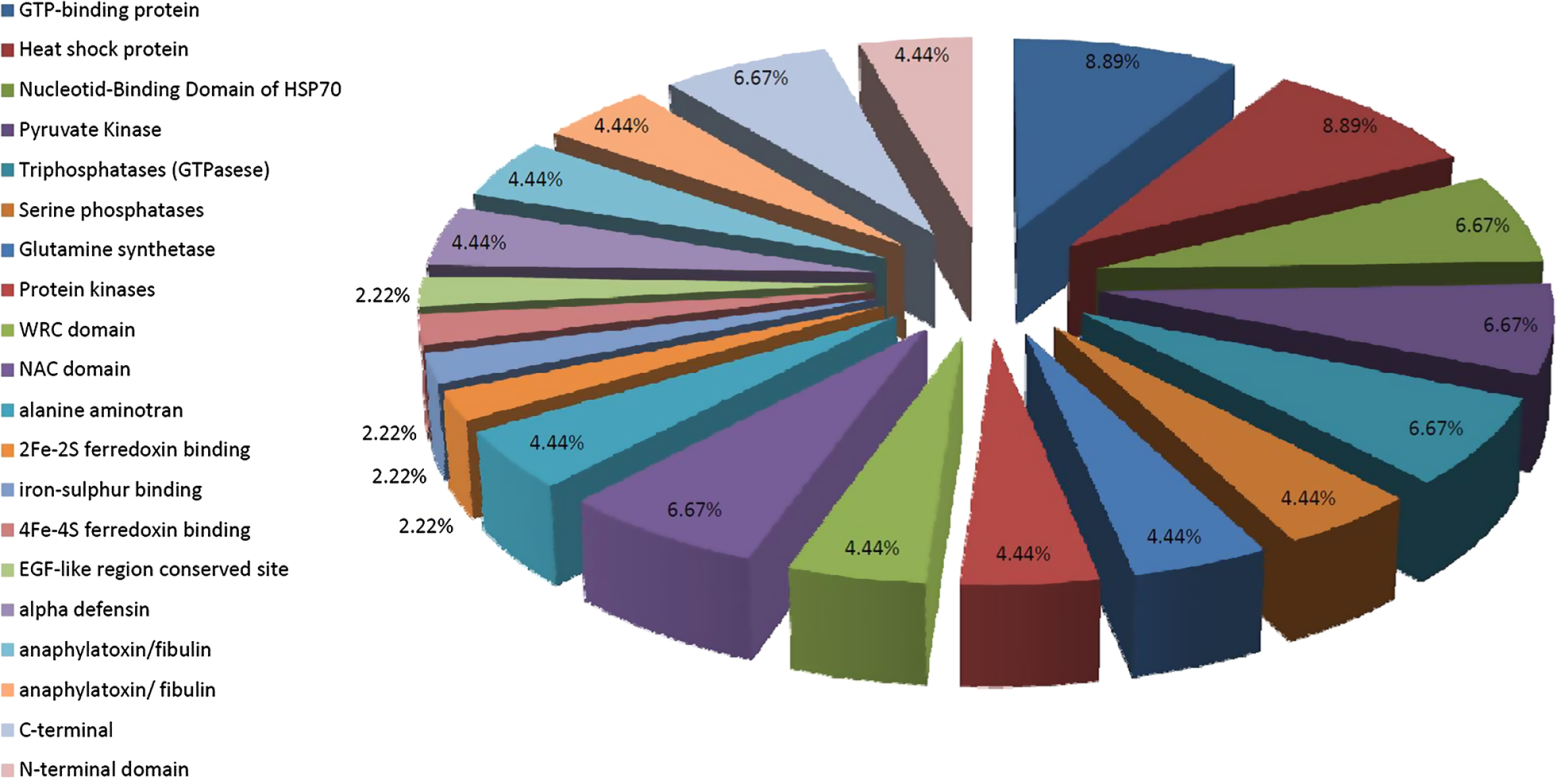
Functional domain marker (FDM) analysis of identified EST-SSRs in the different *Prunus* species assayed

A total of 316 SSR containing sequences were analyzed for FDMs. Mono-nucleotide SSR-containing sequences were not considered for this analysis. Using InterProScan, 3924 functional domains were identified from databases such as pattern scan, SignalPHMM, TMHMM, HMMPanther, and FPrintScan. Functional domains were responsible for GTP-binding protein, Heat shock protein, Nucleotide-Binding Domain of HSP70, Pyruvate Kinase, Triphosphatases (GTPases), Serine phosphatases, Glutamine synthetase, Protein kinases, WRC domain, NAC domain, alanine aminotran, 2Fe-2S ferredoxin binding, iron-sulfur binding, 4Fe-4S ferredoxin binding, EGF-like region conserved site alpha defensin, anaphylatoxin/fibulin, anaphylatoxin/fibulin, C-terminal, N-terminal domain, Cys-rich conserved site, Integrin beta subunit, Alpha defensin, Agouti, Thiolase active site and Tubulin conserved site. Signal P domains searched through SignalPHMM were unintegrated. The SSR-FDM provided information regarding the putative functions of transcribed genetic markers (Fig. 2).

To determine the function of SSR-containing sequences, the 316 sequences from which SSRs were mined were annotated against the non-redundant (nr) protein database available at http://www.ncbi.nlm.nih.gov. Of these, annotations were available for 165 (52.21 %) sequences.

The molecular function refers to the activities, such as catalytic or binding activities, that occur at the molecular level. The proteins identified mainly related to ATP/GTP binding (12 EST-SSRs, 7.27 %), Transferase activity (10, 6.06 %), DNA/RNA binding (8, 4.85 %), Protein binding (7, 4.24 %), and Zinc ion binding (5, 3.03 %). A large number of sequences (151, 47.78 %), however, remained unannotated due to the absence of a homolog in the protein sequence database (Additional file 2: Fig. S2).

For functional annotation, EST-SSRs with significant matches were assigned gene ontology terms in the SwissProt database. A biological process is a series of events accomplished by one or more ordered assemblies of molecular functions. In a gamut of biological processes corresponding to EST-SSRs, the most frequent was ‘Response to stress’ (10 EST-SSRs) followed by ‘Response to cadmium ion’ and ‘Oxidation reduction homeostasis’ (15 EST-SSRs) (Additional file 3: Fig. S3). This Additional file 3: Figure S3 demonstrates all biological processes identified for EST-SSRs across the *Prunus* species assayed.

**Fig. 3:**
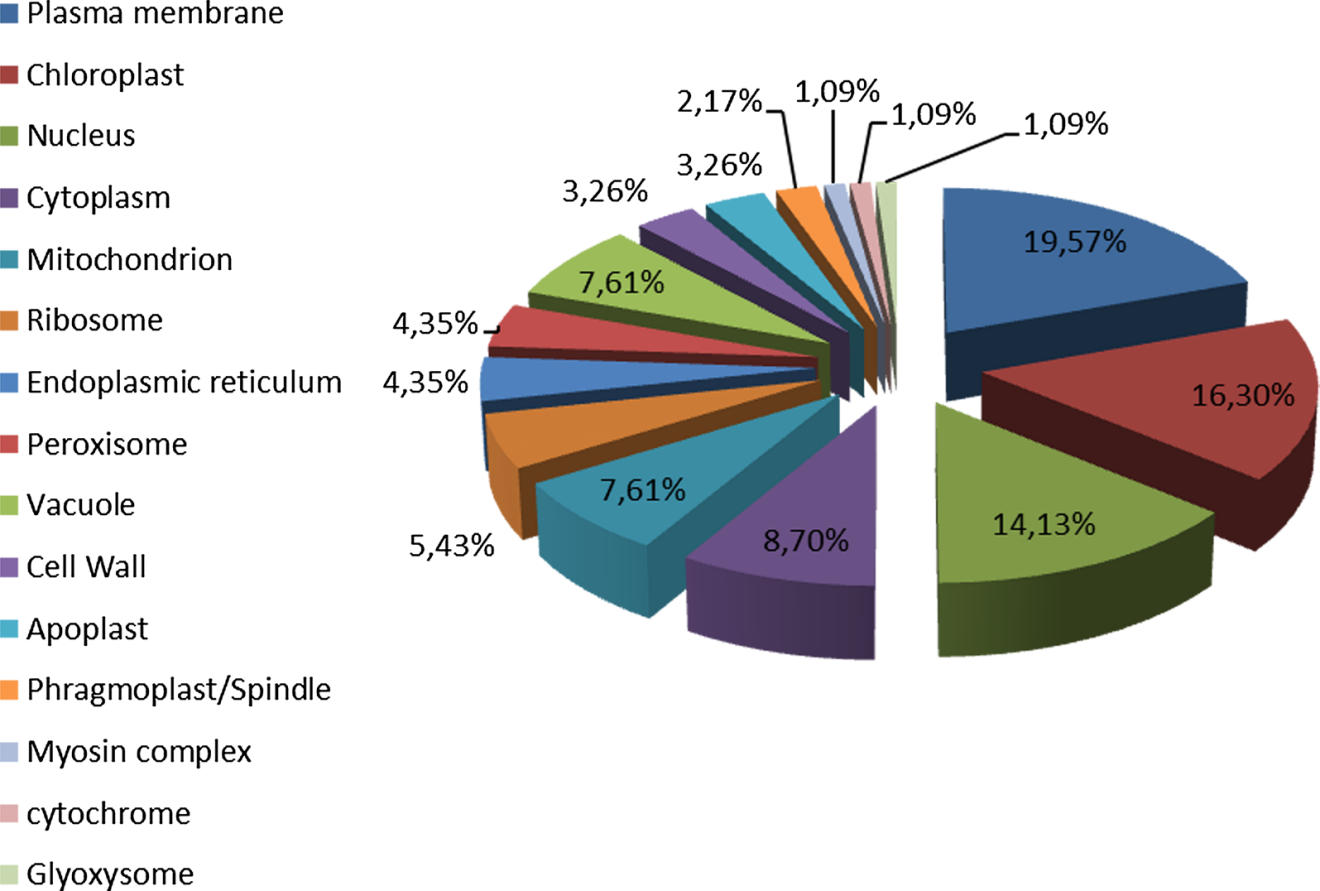
Cellular component of identified EST-SSRs in the different *Prunus* species assayed

Finally, a cellular component represents a component of a cell that it is part of some larger object, e.g., an anatomical structure or a gene product group. In a gamut of cellular components housing putative proteins, the most frequent was ‘Plasma membrane’ (18 EST-SSRs, 19.57 %) followed by ‘Chloroplast’ (15 EST-SSRs, 16.3 %), ‘Nucleus’ (13 EST-SSRs, 14.13 %), and ‘Cytoplasm’ (8 EST-SSRs, 8.7 %) (Fig. 3).

### SSR primer design, prediction of open reading frames and validation in almond and apricot

Of 316 SSRs detected, it was possible to design primers for 175 (55.37 %), whereas acceptable primers could not be produced for the remaining 141 (44.62 %) sequences. The 175 SSRs for which primers were designed were identified across *P. armeniaca* (PruArest SSRs, 21), *P. avium* (PruAvest SSRs, 32), *P. cerasus* (PruCest SSRs, 4), *P. dulcis* (PruDest SSRs, 6), *P. mume* (PruMrest SSRs, 27) and *P. persica* (PruPest, 86), and represented 134 di-, 33 tri-, 2 tetra-, 5 penta- and 1 hexanucleotide repeats. Accession numbers of EST-SSR sequences of *Prunus* species, repeat motifs of SSRs for which primers were designed, primer sequences, product size, and annealing temperatures are provided in Additional file 4: Table S1.

An attempt was made to predict ORFs in SSR containing sequences using ORF Finder. Of the 316 SSRs identified, the positions of 302 SSRs with respect to ORF were determined, whereas no ORF was predicted for the remaining 14 SSR containing sequences. Of these 302 SSRs, 164 (54.30 %) were present in the 5′ UTR, 118 (39.07 %) in ORFs, and the remaining 20 (6.62 %) occurred in the 3′ UTR.

Additional file 4: Table S1 also showed the characterization of these EST-SSRs using the available peach and mei reference genomes. Position and associated genes with respect to the reference genomes of mei and peach has been added. 72 % of developed EST-SSRs were located in the mei reference genome (http://prunusmumegenome.bjfu.edu.cn/) including 85 % of those developed in *P. armeniaca* (PruArest SSRs), 44 % of *P. avium* (PruAvest SSRs), 100 % of *P. cerasus* (PruCest SSRs), 67 % of *P. dulcis* (PruDest SSRs, 6), 90 % of *P. mume* (PruMrest SSRs) and 74 % of those developed in *P. persica* (PruPest, 86). Percentage of EST-SSR located in the peach reference genome (https://www.rosaceae.org/) was lower (35 %) including 0 % of PruArest SSRs, 0 % of PruAvest SSRs, 50 % of PruCest SSRs, 66 % of PruDest SSRs, 33 % of PruMrest SSRs and 60 % of those developed in *P. persica* (PruPest).

On the other hand, 38 SSR markers were validated across peach, almond, plum, pollizo plum and apricot genotypes (Table 2). Results showed a higher transferability level of EST-SSR developed in *P. mume* (PruMrest SSRs) in comparison with the rest of species analyzed. On average the percentage of EST-SSR amplified in the assayed *Prunus* species was of 83.3 % of PruMrest SSR markers, followed by a 62.7 % (PruArest SSRs), 55 % (PruCest SSRs), 40 % (PruPest SSRs), 37.3 % (PruDest SSRs) and 16.6 % (PruAvest SSRs). Differences of success of the total developed EST-SSRs in the assayed *Prunus* species were lesser between 43.6 % in pollizo plum to 58.3 % in almond.

**Table 2:** Percentage of EST-SSR markers amplified in the genotypes assayed from the different studied species

Group	Species	EST-SSR		% of EST-SSR amplification					
of SSR		Identified	Validated	*P. dulcis* (almond)	*P. armeniaca* (apricot)	*P. persica* (peach)	*P. salicina* (plum)	*P. insititia* (pollizo plum)	*Mean*
PruArest	*P. armeniaca*	20	6	83.3	66.6	83.3	66.6	13.8	*62.7*
PruAvest	*P. avium*	32	6	16.6	16.6	16.6	16.6	16.6	*16.6*
PruCest	*P. cerasus*	4	4	50.0	50.0	50.0	50.0	75.0	*55.0*
PruDest	*P. dulcis*	6	6	66.6	33.3	50.0	33.3	33.3	*37.3*
PruMest	*P. mume*	27	6	83.3	83.3	83.3	83.3	83.3	*83.3*
PruPest	*P. persica*	86	10	40.0	30.0	40.0	30.0	30.0	*40.0*
Total		175	38						
*Mean*				*58.3*	*46.3*	*56.5*	*48.3*	*43.6*	

Additional file 5: Table S2 shows the size of 38 SSRs obtained in the analysis of samples of *Prunus* species assayed. All *Prunus* genotypes presented different fingerprints for six of the tested SSRs. No amplification was observed for 14 SSRs assayed during this study. In addition, in two cases these SSRs (PruCest-3 and PruPest-73) only showed amplification in certain species or even in some genotypes inside each species in the case of the EST-SSRs PruArest-1, PruArest-12, PruAres-13, PruArest-15 and PruMest-6. Finally, the level of polymorphism observed ranged from three to ten alleles.

## Discussion

The frequency of SSRs was 8.32 % in assembled sequences, suggesting that *Prunus* species’ ESTs contain relatively high numbers of SSRs. The frequency of SSRs in EST datasets has previously reported as 2.4 % for Arabidopsis, 4.1 % for almond and peach, and 4.8 % for rose [36]. The combined raspberry unigene dataset has 418 contigs and 1671 singletons, from a total of 2089 unigenes [37].

The percentage of SSRs in tissue specific ESTs of some medicinal plants responsible for secondary metabolite production are 4.5 % in *Papaver somniferum*, 10 % in *Phaseolus vulgaris*, 10.8 % in *Coptis japonica*, 12.9 % in *Catharanthus roseus*, and 12.31 % in *Mentha piperita* [38]. The results of the present study are thus in agreement with the previous findings for *Citrus sinensis* (Rutaceae) ([19]), Arabidopsis ESTs [39], and exons of genomic DNA sequences in all eukaryotes studied [40].

Total numbers of SSRs identified in the genomes ranged from 0 to 13,514, with the density of microsatellites ranging from 0 to 7.51 SSRs per Kb. The *P. domestica* genome contained no SSRs, whereas *P. persica* had the most abundant SSRs (13,514). The density of microsatellites was 1.104, 0.647, 1.684, 0.307, and 0.003 SSRs per Kb for *P. avium*, *P. mume*, *P. dulcis*, *P. armeniaca,* and *P. cerasus*, respectively. An average frequency of 1.61 SSRs per Kb was observed, higher than previously reported for some cereal species (1.36 SSRs per Kb) [41], Solanaceae species (1.26 cpSSRs per Kb) [42], *Solanum lycopersicum* (1.3 SSRs per Kb) [18], and *Olea* species (1.47 SSRs per Kb) [29]. In contrast, the average frequency of SSRs identified by the present study from *Prunus* species was lower than observed in loblolly pine (42.9 SSRs per Kb) [28], some cereal species (6 SSRs per Kb) [17], and palms (4.4 SSRs per Kb) [43]. Differences in the frequencies of SSRs between this and previous studies may have been due to differences in the quantity of data analyzed, although it is generally recognized that the abundance of different repeats can vary broadly depending upon the species examined [40]. A study of five different plant species genomes (*A. thaliana*, rice, soybean, maize and bread wheat) revealed that the densities of SSRs in transcribed regions were generally higher than those in genomic DNA [33]. In view of this, future studies should examine the significance of intraspecific variation in the densities of SSRs from different genome regions and interspecific variability across the entire genomes of different plant species [44].

The abundance of different repeat motifs (1–6 bp) in SSRs detected from *Prunus* species during the present study was variable, such that SSRs with different repeat motifs were not evenly distributed. SSRs with dinucleotide repeats (26.31 %) were most abundant, in agreement with the results of earlier studies on Arabidopsis [38]. Similarities may reflect the inclusion of SSRs in non-coding regions of Arabidopsis as well. Smaller repeat motifs were found to be dominant among SSRs identified during this study, with the occurrence of motifs decreasing with increasing repeat lengths. This is consistent with earlier studies conducted [45]. Trinucleotide repeats have previously been found to be abundant in crops [15, 39, 46, 47], as well as citrus [12]. The abundance of trinucleotide SSRs may be attributed to absence of frame shift mutations due to variation in trinucleotide repeats [48]. In the raspberry, trimers, i.e. 3-bp repeats, are more common in gene-coding regions [37].

It was possible to successfully design primers for a very large number (175, 55.37 %) of SSRs during the present study (Additional file 4: Table S1). However, it was not possible to design primers for the remaining SSRs (165, 52.21 %), as the length of sequences flanking both ends of the SSRs was inadequate for primer design. The numerous primer pairs designed during this study can be utilized for a variety of purposes, e.g., gene tagging, genetic mapping, and population studies [37].

In the present study, homologs of 316 SSR containing sequences identified, of which 165 were annotated and categorized into functional classes of protein In Arabidopsis, functions for only 57 % of gene sequences have been assigned, which represents relatively good annotation of sequences, but is still inadequate. Most of the SSR containing sequences that were assigned functions during the present study represented housekeeping genes.

In a previous study, the unigene dataset was aligned to the Gene Ontology (GO) database and classified according to three basic categories: biological process, molecular function, and cellular component. The most abundant GO category was biological process, with a total of 708 sequences associated with metabolic processes, cellular processes, and single organism processes. GO assignments for the molecular function category totaled 323 sequences, with functions for catalytic activity (148), binding (128), and structural molecule activity (47) identified in the raspberry [37]. Additionally, BLAST comparison of the 2089 unigenes to the non-redundant (nr) protein database of NCBI yielded 1664 matches (80 %) [37].

The new EST-SSRs identified during the present study enlarge the number of EST-SSRs identified in *Prunus* species, including the 256 identified in peach [4, 5, 14], the 34 identified in apricot [6, 7], the 29 identified in almond [8, 9], and the 24 identified previously mei [10, 11]. Only for the peach, were 52 of these EST-SSRs previously identified [13]. These authors identified using in silico search around 15,000 EST-SSR inside the peach reference genome [13].

The characterization of these EST-SSRs using the available peach and mei reference genomes showed a higher synteny level and positioning of markers in the mei reference genome. In agreement with these results, EST-SSR validation also showed a higher transferability level of EST-SSR developed in *P. mume* (mei) in comparison with the rest of species analyzed indicating a higher level of synteny. This result should also indicate the better suitability of its reference genome in comparison with the peach genomes for the wide use in *Prunsu* species.

Acceptable PCR primers were designed for 175 simple sequence repeats (SSRs) out of 316 identified SSRs using default settings in the Primer3 software. However, the success rate for the PCR primer design in the different *Prunus* species assayed is quite moderate (about 55 %). For this reason an alternative to develop better SSR marker should be to design PCR primers with less stringent parameter settings in Primer3 or to use another PCR primer design software. Transferability rates, however, are in accordance with the described phylogenetic characterization [1] of the assayed species being peach and almond from the subgenus *Amygdalus*, sweet and sour cherry from the subgenus *Cerasus*, plum and pollizo plum from the subgenus *Cerasus* section Prunus, and apricot and mei from the subgenus *Cerasus* section Armeniaca (Additional file 6: Fig. S4).

Cross amplification of the SSRs developed from *Prunus* species offers new functional genomic opportunities given the well-known synteny among *Prunus* genomes [36] and transcriptomes [49]. However, no amplification was observed for some SSRs assayed during this study, indicating the limitation of transferability of all EST-SSR markers across the *Prunus* genus. In addition, the low polymorphism observed should be due to the reduced number of genotypes assayed in each species. EST-SSR validation also showed a higher transferability level of EST-SSR developed in *P. mume* (mei) in comparison with the rest of species analyzed indicating a higher level of synteny.

Our results confirm the suitability of EST-SSR markers for cultivar discrimination and assessment of genetic diversity and clustering in apricot, as has been previously demonstrated for apricot, peach, and cherry. In addition, we have demonstrated that the EST-SSR markers developed are of great utility in the taxonomic characterization of different species.

The use of coding DNA regions for SSR development represents an additional advantage in association genetic [50] and linkage analysis, as gene functions are often known [51]. Recently, three EST-SSRs developed from flavonoid pathway transcription factors have been assayed as markers for fruit color selection in Japanese plum breeding programs [52].

## Conclusions

Development and application of molecular markers is of immense importance in the examination of the genetic composition, inter-species variability, and evolutionary relationships of *Prunus* species. EST-SSRs developed by the present study provide significant insight into these areas. This study demonstrates an approach to develop computationally mined SSRs from ESTs. Derived SSRs can be used in related species for which less sequence data is available, given the high interspecific transferability of EST-SSRs, thus enhancing cross species attempts to develop conserved orthologous marker sets. The use of coding DNA regions for SSR development represents an additional advantage as gene functions are often known. Findings will aid analysis of functionally important molecular markers and facilitate the analysis of genetic diversity. In addition, these SSRs developed here can be used as molecular markers linked to genes of agronomic interest in association genetic studies and quantitative trait locus (QTL) analysis.

## Methods

### Processing and assembly of EST sequences, and SSR identification and characterization

All EST sequences of *Prunus* species, namely peach (*P. persica*), apricot (*P. armeniaca*), sweet cherry (*P. avium*), mei (*P. mume*), almond (*P. dulcis*), sour cherry (*P. cerasus*) and prune (*P. domestica*) were downloaded from Genbank (ftp://ncbi.nlm.nih.gov/genbank/genomes/). To construct longer and less redundant sequences, publicly available ESTs were assembled from CAP3 [16]. CAP3 is a commonly used program [53, 54] that identifies overlapping sequences and generates contigs with consensus sequences. The objective was the elimination of redundancy in EST sequences to arrive at a contiguous sequence (contigs) that can be used for analysis of SSRs. For the purpose of SSR identification, CAP3 contig and singleton outputs were combined to form non-redundant sequence data. Genomic SSRs were detected using GMATo (http://sourceforge.net/p/GMATo) (Additional file 7: Fig. S5). The minimum length of SSR was fixed at 14 bp in accordance with criteria used by [14]. SSRs were defined as ≥14 bp mononucleotide or dinucleotide repeats; ≥15 bp trinucleotide repeats; ≥16 tetranucleotide repeats; ≥20 pentanucleotide repeats; and ≥18 hexanucleotide repeats.

### Functional domain marker (FDM) analysis

Functional domain markers (FDMs) were found from SSR containing sequence using InterProScan at EMBL-EBI (http://www.ebi.ac.uk/interpro/search/sequence-search). InterProScan provides the platform to analyze functional domains with the help of member databases, such as BlastProDom, FPrintScan, HMMPIR, HMMPfam, HMMSmart, HMMTigr, ProfileScan, HAMAP, PatternScan, SuperFamily, SignalPHMM, TMHMM, HMMPanther, and Gene3D. EST-SSR sequences were searched for significant matches using BLASTx against non-redundant protein database entries (http://blast.ncbi.nlm.nih.gov/Blast.cgi). BLASTx searches protein databases using a translated nucleotide query. BLASTx was performed at identity >70 %. SSR-FDM contig sequences determined from Interproscan were annotated for biological processes, cellular components, and molecular functions using the QuickGO browser for Gene Ontology terms and annotation.

### SSR primer design, prediction of open reading frames and characterization using reference genomes

Primer design for EST-SSR sequences was performed using Primer3 with default parameters: optimum primer size = 20.0 (range of 18–27), optimum annealing temperature = 60.0 (range of 57.0–63.0), GC content of 20–80 %. Open reading frames (ORFs) were predicted for all SSR containing sequences using the ORF Finder available at NCBI using standard genetic code. Sequence fragments corresponding to the maximum length uninterrupted by a stop codon were taken as the primary encoding segment (ORF) of query sequences. In all predicted ORFs, the relative position of SSRs was detected, i.e., whether the SSR was present within the ORF, in the 5′ or 3′ un-translated region (UTR) [19]. Using Primer-BLAST, SSRs were also characterized (in terms of position in the reference genome and associated gene) using the two available *Prunus* reference genomes for mei (http://prunusmumegenome.bjfu.edu.cn/) [55] and peach (https://www.rosaceae.org/) [56].

### Validation of EST-SSR markers in different *Prunus* genotypes

Plant material used for validation assay analysis consisted of 16 *Prunus* genotypes from different species including almond (‘Antoñeta’, ‘D0-078’, ‘Marcona’, and ‘Ferragnés’), apricot (‘Rojo Pasión’, ‘Z506-7’, ‘Currot’, ‘Orange Red’ and ‘Goldrich’), peach (‘GF-305’ and ‘Baby Gold-6’), plum (‘Golden Kiss’, ‘Larry Anne’ and ‘Saphire’) and pollizo plum (*P. inisitia*) (‘PS2’ and ‘Adesoto 101’) (Additional file 2: Table S2). Total DNA was isolated using the procedure previously described by Doyle and Doyle [57]. Approximately 50 mg of young leaves were ground in a 1.5-ml Eppendorf tube with 750 μl of CTAB extraction buffer (100 mM Tris–HCl, 1.4 M NaCl, 20 mM EDTA, 2 % CTAB, 1 % PVP, 0.2 % mercaptoethanol, 0.1 % NaHSO_3_). Samples were incubated at 65 °C for 20 min, mixed with an equal volume of 24:1 chloroform-isoamyl alcohol, and centrifuged at 6000*g* for 20 min. The upper phase was recovered and mixed with an equal volume of isopropanol at −20 °C. The nucleic acid precipitated was washed in 400 μl of 10 mM NH_4_Ac in 76 % ethanol, dried, resuspended in 50 μl of TE (10 mM Tris–HCl, 0.1 mm EDTA, pH 8.0), and incubated with 0.5 μg of RNase A at 37 °C for 30 min, to digest RNA.

Extracted genomic DNA was PCR-amplified using 40 primer pairs of the identified EST-SSRs. SSR-PCR reactions were performed in a 25 μl volume using the protocol described by Sánchez-Pérez et al. [58]. The reaction mixture contained 16 mM (NH4)_2_SO_4_, 67 mM Tris–HCl pH 8.8, 0.01 % Tween 20, 2 mMMgCl2, 0.2 mM of each primer, 0.1 mM of each dNTP, one unit of Eco-Taq DNA Polymerase (Ecogen S.R.L., Barcelona, Spain), and 90 ng of genomic DNA. Amplification was performed for 40 cycles at 94 °C for 30 s, 58 °C for 1 min 30 s, and 72 °C for 1 min, for denaturation, annealing, and primer extension, respectively. Finally, amplified PCR products were separated by electrophoresis using 3 % Metaphor^®^ agarose gel (Biowittaker, Maine, USA) (1 X TBE buffer) stained with GelRed™ Nucleic Acid Gel Stain^®^ (Biotium, Hatwad, CA, USA). A 1 Kb Plus DNA Ladder was used as molecular size standard. Band scoring was analyzed using SYNGENE^®^ GeneTools gel analysis software (Cambridge, UK).

## Additional files

10.1186/s13104-016-2143-y Percentage frequency of polar and non-polar amino acids in *Prunus* species: *Prunus armeniaca* (a), *Prunus avium* (b), *Prunus persica* (c), *Prunus cerasus* (d), *Prunus dulcis* (e), and *Prunus mume* (f).
10.1186/s13104-016-2143-y Molecular Function of identified EST-SSRs in the different *Prunus* species assayed.
10.1186/s13104-016-2143-y Biological Process of identified EST-SSRs in the different *Prunus* species assayed.
10.1186/s13104-016-2143-y Description of SSR containing ESTs with significant matches in *Prunus* species, including accession numbers, repeat motif, primer sequences, product size, and annealing temperature. In addition, position and associated genes with respect to the reference genomes of mei and peach has been added. EST-SSR marked by asterisk has been previously described by Chen et al. [13].
10.1186/s13104-016-2143-y Allele sizes of the 38 SSRs assayed in the analysis of peach, almond, plum, pollizo plum and apricot genotypes. na = no amplification.
10.1186/s13104-016-2143-y Phylogenetic characterization of the assayed species described by Potter [1].
10.1186/s13104-016-2143-y Work flow chart.

## References

[1] Potter D, Kole C, Abbott AG (2012). Basic information on the stone fruit crops. Genetics, genomics and breeding of stone fruits.

[2] Kantety RV, La Rota M, Matthews DE, Sorrells ME (2002). Data mining for simple sequence repeats in expressed sequence tags from barley, maize, rice, sorghum and wheat. Plant Mol Biol.

[3] Nagaraj SH, Gasser RB, Ranganathan S (2007). A hitchhiker’s guide to expressed sequence tag (EST) analysis. Brief Bioinform..

[4] Yamamoto T, Mochida K, Imai T, Shi IZ, Ogiwara I, Hayashi T (2002). Microsatellite markers in peach [*Prunus persica* (L.) Batsch] derived from an enriched genomic and cDNA libraries. Mol Ecol Notes.

[5] Vendramin E, Dettori MT, Giovinazzi J, Micali R, Quarta R, Verde I (2007). A set of EST-SSRs isolated from peach fruit transcriptome and their transportability across Prunus species. Mol Ecol Notes.

[6] Decroocq V, Favé MG, Hagen L, Bordenave L, Decroocq S (2003). Development and transferability of apricot and grape EST microsatellite markers across taxa. Theor Appl Genet.

[7] Hagen LS, Chaib J, Fad B, Decrocq V, Bouchet P, Lambert P (2004). Genomic and cDNA microsatellite from apricot (*Prunus armeniaca* L). Mol Ecol Notes.

[8] Xu Y, Ma RC, Xie H, Liu JT, Cao MQ (2004). Development of SSR markers for the phylogenetic analysis of almond trees from China and the Mediterranean region. Genome..

[9] Xie H, Sui Y, Chang FQ, Xu Y, Ma RC (2006). SSR allelic variation in almond (*Prunus dulcis* Mill). Theor Appl Genet..

[10] Li X, Shangguan L, Song C, Wang S, Gao Z, Yu H (2014). Analysis of expressed sequence tags from *Prunus mume* flower and fruit and development of simple sequence repeat markers. BMC Genet.

[11] Wang YJ, Li XY, Han J, Fang WM, Li XD, Wang SS (2014). Analysis of genetic relationships and identification of flowering-mei cultivars using EST-SSR markers developed from apricot and fruiting-mei. Scientia Hort..

[12] Chen C, Zhou P, Choi YA, Huang S, Gmitter FG (2006). Mining and characterizing microsatellites from citrus ESTs. Theor Appl Genet.

[13] Chen C, Bock CH, Okie WR, Gmitter FG, Jung S, Main D (2014). Genome-wide characterization and selection of expressed sequence tag simple sequence repeat primers for optimized marker distribution and reliability in peach. Tree Genet Gen..

[14] Dettori MT, Micali S, Giovinazzi J, Scalabrin S, Verde I, Cipriani G (2015). Mining microsatellites in the peach genome: development of new long-core SSR marker for genetic analyses in five *Prunus* species. Springerplus.

[15] Gupta PK, Rustgi S, Sharma S, Singh R, Kumar N, Balyan HS (2003). Transferable EST-SSR markers for the study of polymorphism and genetic diversity in bread wheat. Mol Genet Genomics.

[16] Huang X, Madan A (1999). CAP3: a DNA sequence assembly program. Genome Res.

[17] Varshney RK, Thiel T, Stein N, Langridge P, Graner A (2002). In silico analysis on frequency and distribution of microsatellites in ESTs of some cereal species. Cell Mol Biol Lett.

[18] Gupta S, Shukla R, Roy S, Sen N, Sharma A (2010). In silico SSR and FDM analysis through EST sequences in *Ocimum basilicum*. Plant Omics J..

[19] Shanker A, Bhargava A, Bajpai R, Singh S, Srivastava S, Sharma V (2007). Bioinformatically mined simple sequence repeats in UniGene of *Citrus sinensis*. Sci Horti..

[20] Poncet V, Rondeau M, Tranchant C, Cayrel A, Hamon S, de Kochko A (2006). SSR mining in coffee tree EST databases: potential use of EST-SSRs as markers for the Coffea genus. Mol Genet Genomics..

[21] Aggarwal RK, Hendre PS, Varshney RK, Bhat PR, Krishnakumar V, Singh L (2007). Identification, characterization and utilization of EST-derived genic microsatellite markers for genome analyses of coffee and related species. Theor Appl Genet.

[22] Pinto LR, Oliveira KM, Ulian EC, Garcia AAF, de Souza AP (2004). Survey in the sugarcane expressed sequence tag database (SUCEST) for simple sequence repeats. Genome..

[23] Pashley CH, Ellis JR, McCauley DE, Burke JM (2006). EST databases as a source for molecular markers: lessons from *Helianthus*. J Hered.

[24] Heesacker A, Kishore VK, Gao W, Tang S, Kolkman JM, Gingle A (2008). SSRs and INDELs mined from the sunflower EST database: abundance, polymorphisms, and cross-taxa utility. Theor Appl Genet.

[25] Thiel T, Michalek W, Varshney RK, Graner A (2003). Exploiting EST databases for the development and characterization of gene derived SSR-markers in barley (*Hordeum vulgare* L.). Theor Appl Genet.

[26] Yu JK, Dake TM, Singh S, Benscher D, Li W, Gill B (2004). Development and mapping of EST-derived simple sequence repeat markers for hexaploid wheat. Genome..

[27] Acuña CV, Fernández P, Villalba PV, García MN, Hopp HE, Poltri SNM (2012). Discovery, validation, and in silico functional characterization of EST-SSR markers in *Eucalyptus globules*. Tree Genet Gen..

[28] Bérubé Y, Zhuang J, Rungis D, Ralph S, Bohlmann J, Ritland K (2007). Characterization of EST-SSRs in loblolly pine and spruce. Tree Genet Gen..

[29] Filiz E, Koc I (2012). In silico chloroplast SSRs mining of *Olea* species. Biodiversitas..

[30] Qiu L, Yang C, Tian B, Yang JB, Liu A (2010). Exploiting EST databases for the development and characterization of EST-SSR markers in castor bean (*Ricinus communis* L.). BMC Plant Biol.

[31] Yu JK, Paik H, Choi JP, Han JH, Choe JK, Hur CG (2010). Functional domain marker (FDM): and in silico demonstration in *Solanaceae* using simple sequence repeats (SSR). Plant Mol Biol Rep..

[32] Filiz E (2013). SSRs mining of Brassica species in mitochondrial genomes: bioinformatic approaches. Hort Environ Biotech..

[33] Morgante M, Hanafey M, Powell W (2002). Microsatellites are preferentially associated with nonrepetitive DNA in plant genomes. Nat Genet.

[34] Andersen JR, Lubberstedt T (2003). Functional markers in plants. Trends Plant Sci.

[35] Varshney RK, Graner A, Sorrells ME (2005). Genic microsatellite markers in plants: features and applications. Trends Biotech..

[36] Jung S, Jesudurai C, Staton M, Du Z, Ficklin S, Cho I (2004). GDR (genome database for rosaceae): integrated web resources for rosaceae genomics and genetics research. BMC Bioinformatics..

[37] Bushakra JM, Lewers KS, Staton ME, Zhebentyayeva T, Saski CA (2015). Developing expressed sequence tag libraries and the discovery of simple sequence repeat markers for two species of raspberry (*Rubus* L.). BMC Plant Biol.

[38] Tripathi KP, Roy S, Khan F, Shasany AK, Sharma A, Khanuja SPS (2008). Identification of SSR-ESTs corresponding to alkaloid, phenylpropanoid and terpenoid biosynthesis in MAP’s. Online J Bioinf..

[39] Cardle L, Ramsay L, Milborne D, Macaulay M, Marshall D, Waugh R (2000). Computational and experimental characterization of physically clustered simple sequence repeats in plants. Genetics.

[40] Toth G, Gaspari Z, Jurka J (2000). Microsatellites in different eukaryotic genomes: survey and analysis. Genome Res.

[41] Melotto-passarin DM, Tambarussi EV, Dressano K, De Martin VF, Carrer H (2011). Characterization of chloroplast DNA microsatellites from *Saccharum* spp. and related species. Genet Mol Res.

[42] Tambarussi EV, Melotto-passarin DM, Barbosa AL, Brigati JB (2009). In silico analysis of simple sequence repeats from chloroplast genomes of *Solanaceae* species. Crop Breed Appl Biotech..

[43] Palliyarakkal MK, Ramaswamy M, Vadivel A (2011). Microsatellites in palm (*Arecaceae*) sequences. Bioinformation..

[44] Martínez-Gómez P, Sánchez-Pérez R, Rubio M (2012). Clarifying omics concepts, challenges, and opportunities for Prunus breeding in the postgenomic era. OMICS.

[45] Karaoglu H, Lee CMY, Meyer W (2005). Survey of simple sequence repeats in completed fungal genomes. Mol Biol Evol.

[46] Scott KD, Eggler P, Seaton F, Rossetto M, Ablett EM, Lee LS, Henry RJ (2000). Analysis of SSRs derived from grape ESTs. Theor Appl Genet.

[47] Varshney RK, Kumar A, Balyan HS, Joy KR, Prasad M, Gupta PK (2000). Characterization of microsatellites and development of chromosome specific STMS markers in bread wheat. Plant Mol Biol Report..

[48] Metzgar D, Bytof J, Wills C (2000). Selection against frameshift mutations limits microsatellite expansion in coding DNA. Genome Res.

[49] Martínez-Gómez P, Crisosto CH, Bonghi C, Rubio M (2011). New approaches to *Prunus* transcriptome analysis. Genetica.

[50] Sorkheh K, Malysheva-Otto V, Wirthensohn MG, Tarkesh-Esfahani S, Martínez-Gómez P (2008). Linkage disequilibrium, genetic association mapping and gene localization in crop plants. Genet Mol Biol..

[51] Salazar JA, Ruiz D, Campoy JA, Sánchez-Pérez R, Crisosto CH, Martínez-García PJ (2014). Quantitative Trait Loci (QTL) and Mendelian Trait Loci (MTL) Analysis in *Prunus* A Breeding Perspective and Beyond. Plant Mol Biol Rep..

[52] González M, Salazar E, Castillo J, Morales P, Mura-Jornet I, Maldonado J (2016). Genetic structure based on EST-SSR: a putative tool for fruit color selection in Japanese plum (*Prunus salicina* L.) breeding programs. Mol Breed.

[53] Whitfield W, Band R, Bonaldo F, Kumar G, Liu L, Pardinas R (2002). Annotated expressed sequence tags and cDNA microarrays for studies of brain and behavior in the honey bee. Genome Res.

[54] Pertea G, Huang X, Liang F, Antonescu V, Sultana R, Karamycheva S (2003). TIGR gene indices clustering tools (TGICL): a software system for fast clustering of large EST datasets. Bioinformatics.

[55] Zhang Q, Chen W, Sun L, Zhao F, Huang B, Yang W (2012). The genome of *Prunus mume*. Nat Commun..

[56] Verde I, Abbott AG, Scalabrin S, Jung S, Shu S, Marroni F (2013). The high-quality draft of peach (*Prunus* persica) identifies unique patterns of genetic diversity, domestication and genome evolution. Nat Genetics..

[57] Doyle JJ, Doyle JL (1987). A rapid DNA isolation procedure for small quantities of fresh leaf tissue. Phytochem Bull..

[58] Sánchez-Pérez R, Ballester J, Dicenta F, Arús P, Martínez-Gómez P (2006). Comparison of SSR polymorphisms using automated capillary sequencers, and polyacrylamide and agarose gel electrophoresis: implications for the assessment of genetic diversity and relatedness in almond. Sci Hort..

