# Supplementary figures and images for: In silico search, characterization and validation of new EST-SSR markers in the genus Prunus
Source: BMC Res Notes. 2016 Jul 7;9:336. doi: 10.1186/s13104-016-2143-y (PMC4937603; doi:10.1186/s13104-016-2143-y)

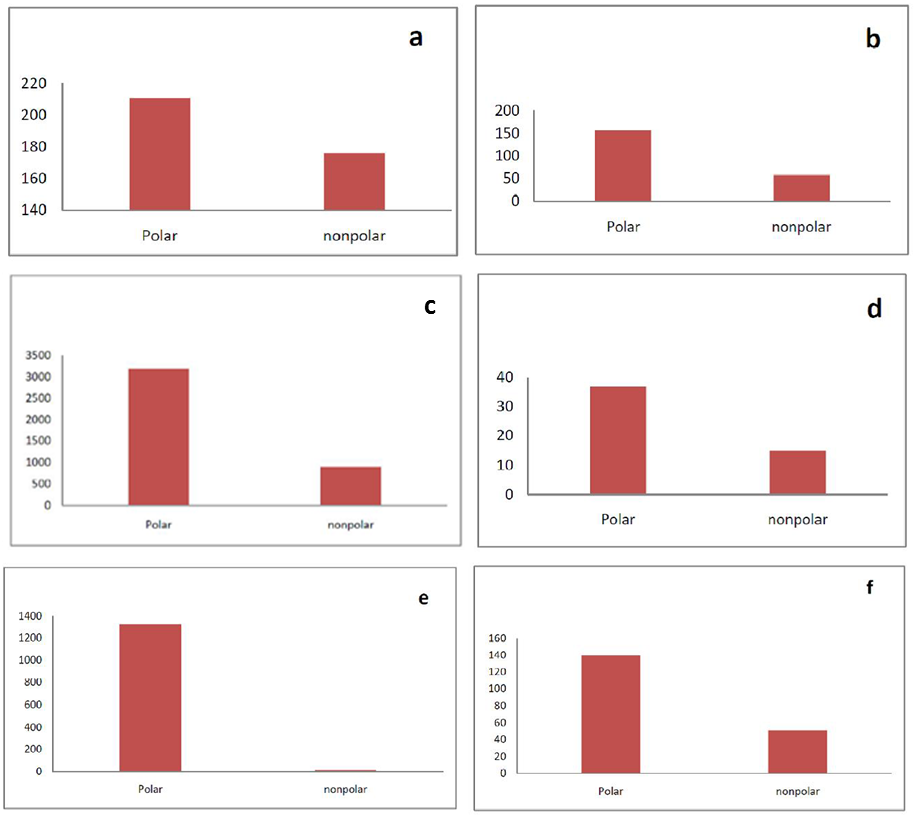

Supplement: Supplementary file 1 — 10.1186/s13104-016-2143-y Percentage frequency of polar and non-polar amino acids in Prunus species: Prunus armeniaca (a), Prunus avium (b), Prunus persica (c), Prunus cerasus (d), Prunus dulcis (e), and Prunus mume (f). [file 13104_2016_2143_MOESM1_ESM.tif]

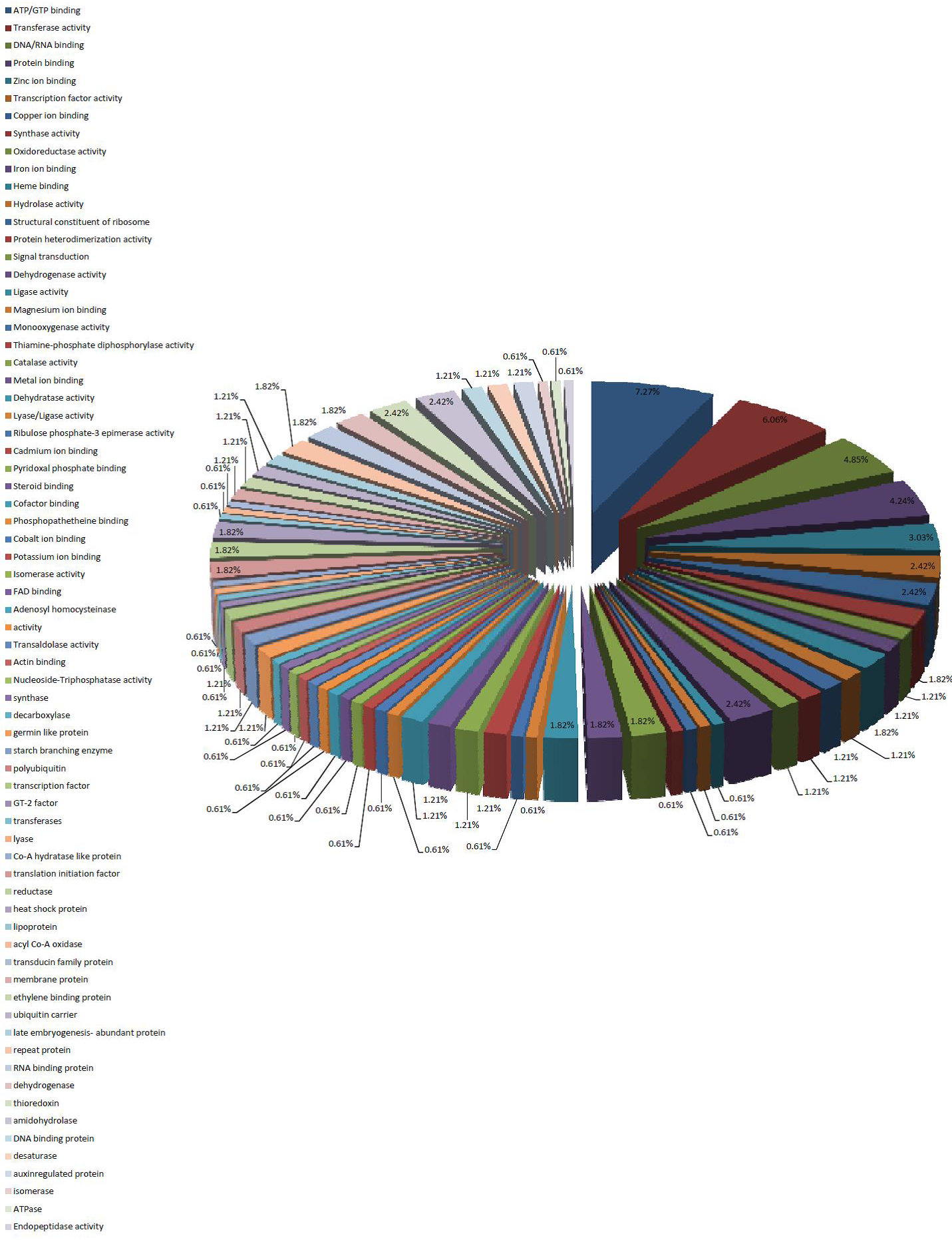

Supplement: Supplementary file 2 — 10.1186/s13104-016-2143-y Molecular Function of identified EST-SSRs in the different Prunus species assayed. [file 13104_2016_2143_MOESM2_ESM.tif]

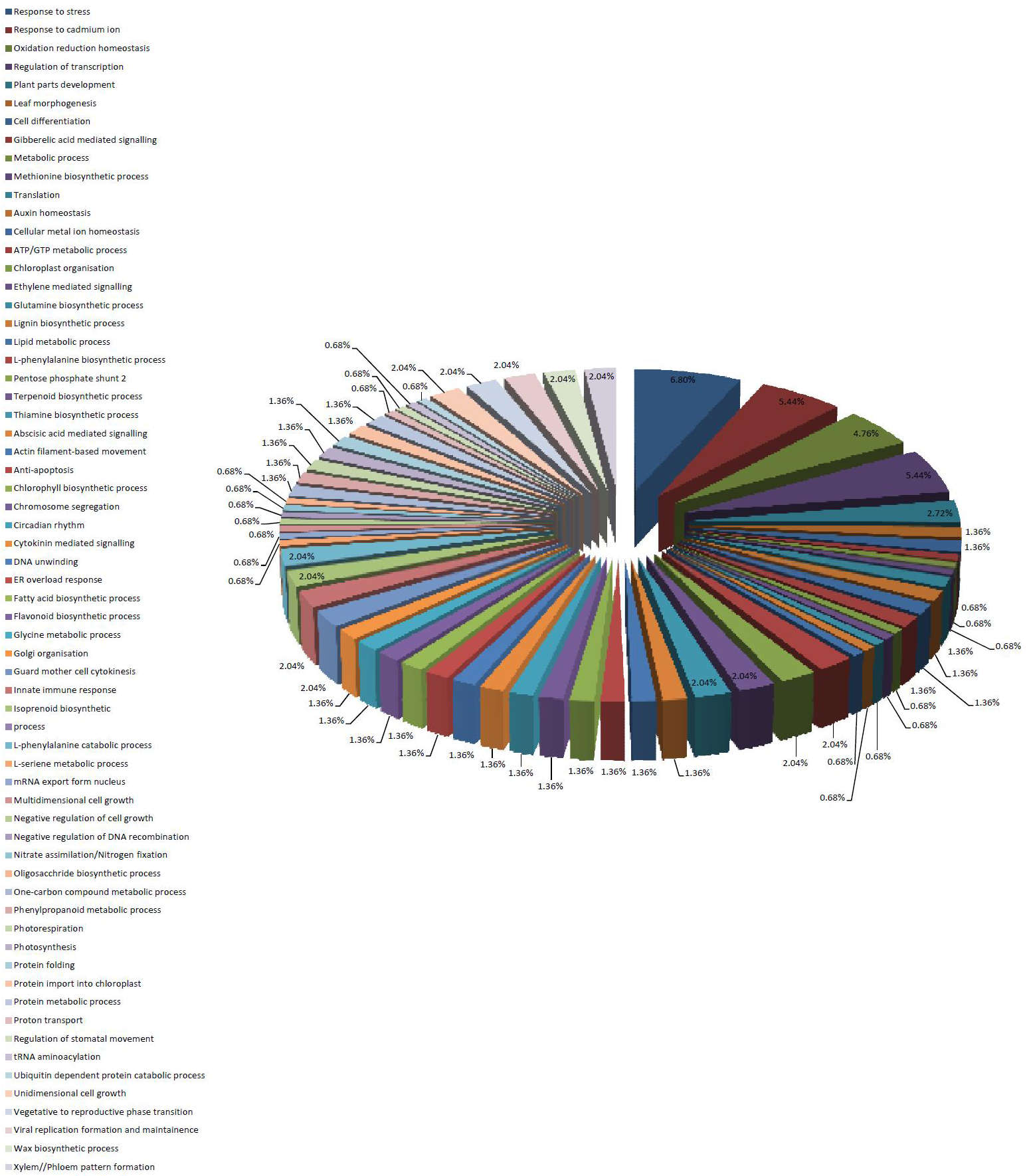

Supplement: Supplementary file 3 — 10.1186/s13104-016-2143-y Biological Process of identified EST-SSRs in the different Prunus species assayed. [file 13104_2016_2143_MOESM3_ESM.tif]

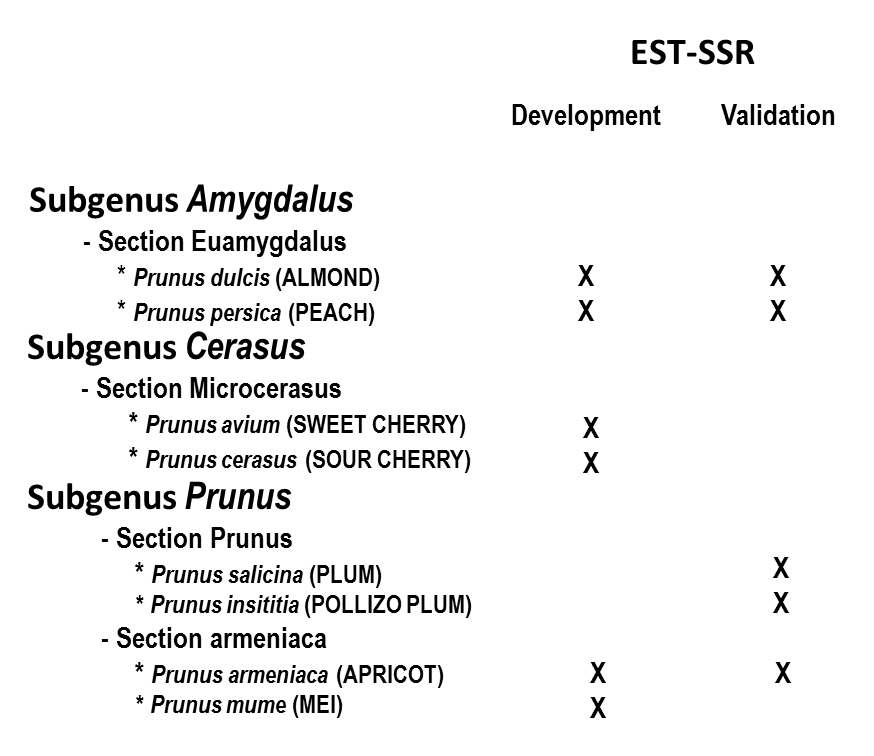

Supplement: Supplementary file 6 — 10.1186/s13104-016-2143-y Phylogenetic characterization of the assayed species described by Potter [1]. [file 13104_2016_2143_MOESM6_ESM.tif]

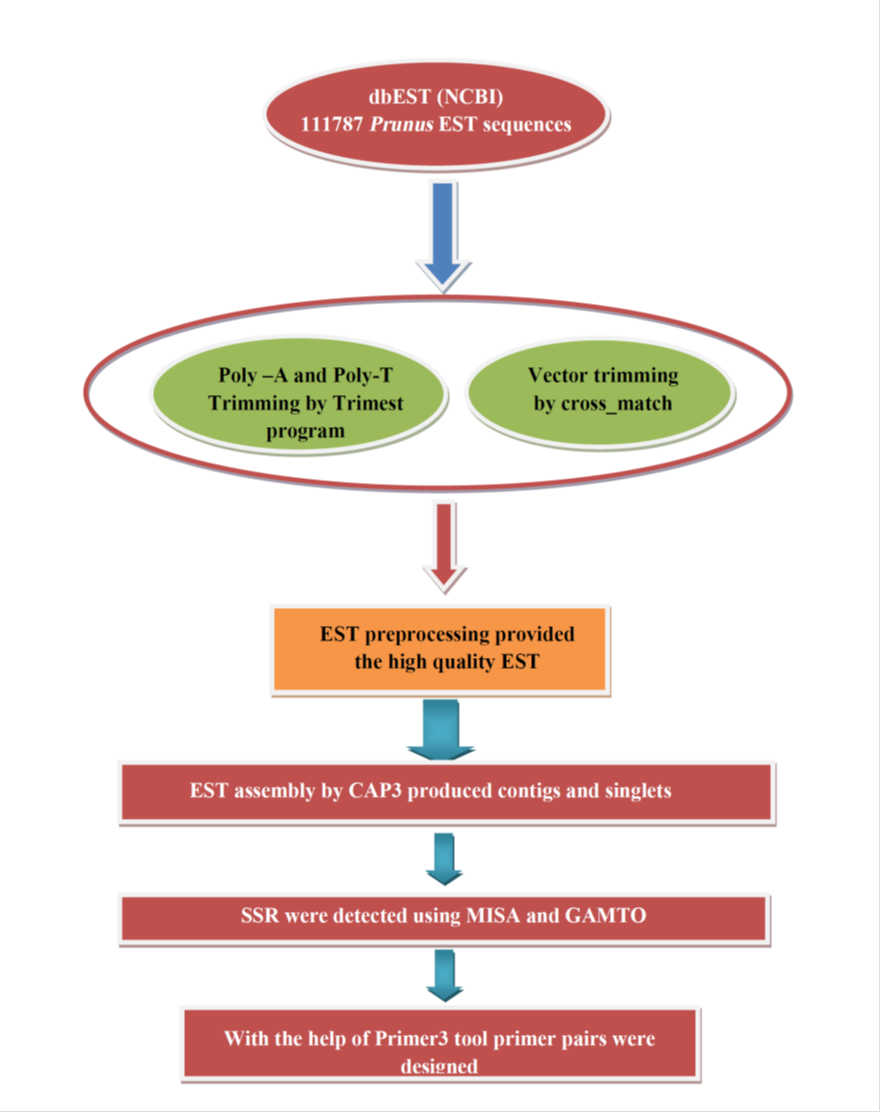

Supplement: Supplementary file 7 — 10.1186/s13104-016-2143-y Work flow chart. [file 13104_2016_2143_MOESM7_ESM.tif]
